# Traditional Tibetan Medicine Twenty-Five Wei’er Tea Pills Ameliorate Rheumatoid Arthritis Based on Chemical Crosstalk Between Gut Microbiota and the Host

**DOI:** 10.3389/fphar.2022.828920

**Published:** 2022-02-10

**Authors:** Zixuan Li, Lijuan Nie, Yong Li, Lu Yang, Lulu Jin, Baozhong Du, Juan Yang, Xulin Zhang, Huantian Cui, Ouzhu Luobu

**Affiliations:** ^1^ Department of Basic Medicine, Medical College of Tibet University, Lhasa, China; ^2^ Department of Pharmacy, Medical College of Tibet University, Lhasa, China; ^3^ Institute of Oxygen Supply, Center of Tibetan Studies (Everest Research Institute), Tibet University, Lhasa, China; ^4^ Tianjin University of Traditional Chinese Medicine, Tianjin, China; ^5^ Second Affiliated Hospital of University of South China, Hengyang, China; ^6^ Shandong Provincial Key Laboratory of Animal Cell and Developmental Biology, School of Life Sciences, Shandong University, Qingdao, China; ^7^ Medical College of Tibet University, Lhasa, China; ^8^ Affiliated Fukang Hospital of Tibet University, Lhasa, China

**Keywords:** Tibetan medicine, twenty-five Wei’er tea pills, rheumatoid arthritis, gut microbiota, metabolomics

## Abstract

Twenty-Five Wei’er Tea Pills (TFP), a traditional Tibetan medicine, has shown to have a promising therapeutic effect in patients with Rheumatoid arthritis (RA), as well as being safe. Nonetheless, there have been limited pharmacological studies that have explored this therapeutic option. As gut microbiota has been proven to have a critical role in the pathogenesis of RA, this study aims to explore and reveal relevant ways by which TFP interacts with the chemical crosstalk between the gut microbiome and its host. 16S rRNA sequencing, combined with un-targeted metabolomics, were conducted on collagen-induced arthritis (CIA) rats. CIA model rats treated with TFP showed significant improvement in weight gain, pathological phenomena in joints, as well as decreased serum levels of TNF-α, IL-6 and increased level of IL-4 and IL-10. Significant dysfunction in the gut microbiome and alteration in serum metabolites were observed in CIA model rats, which were restored by TFP treatment. Coherence analysis indicated that TFP modulated the pathways of histidine metabolism, phenylalanine metabolism, alanine, aspartate, glutamate metabolism, amino sugar and nucleotide sugar metabolism owing to the abundances of *Lactobacillus*, *Bacteroides*, Prevotellaceae*_UCG-001* and Christensenellaceae*_R-7_group* in the gut microflora. The corresponding metabolites involved L-histidine, histamine, phenylethylamine, asparagine, L-aspartic acid, D-fructose 1-phosphate, D-Mannose 6-phosphate, D-Glucose 6-phosphate, and Glucose 1-phosphate. In conclusion, this study reveals the ameliorative effects of TFP on RA through the chemical crosstalk that exists between the gut microbiota and its host, and also further enriches our understandings of the pathogenesis of RA.

## Introduction

Rheumatoid arthritis (RA) is a chronic immune disease, which involves multiple systems. The hallmark pathological features of RA include the proliferation of synovial lining cells, significant inflammatory cell infiltration into the interstitium, regeneration of microvessels, pannus formation, damage of both cartilage and bone tissue, as well as reported metabolic disorders (Liu et al., 2021; Jiang et al., 2021). Globally, the prevalence of RA is relatively high, about 0.5–1.0% (Tobon et al., 2010; Ardalan and Vahed, 2017). This autoimmune disease is primarily diagnosed in patients aged between 40 and 60 years, and is approximately three times more prevalent in women compared with in men(Tobon et al., 2010). Its clinical manifestations are joint stiffness, deformity, rigidity, and even joint dislocation; furthermore, RA has an exceptionally high disability rate. The predominantly prescribed medications to treat RA include nonsteroidal anti-inflammatory drugs (NSAIDs), glucocorticoids (GCs), disease-modifying antirheumatic drugs, and targeted biological agents. However, due to the complex pathogenesis of RA, these drugs have failed to produce satisfactory pharmacological effects while also being known create severe side effects, including immunodeficiency, gastrointestinal disorders, and bodily fluid disorders.

An increasing number of studies have shown the effect of gut microbiota on the immune system (de Oliveira et al., 2017; Castro Rocha et al., 2020), metabolic homeostasis (Wu et al., 2020; Tsai et al., 2021), and gastrointestinal integrity (Owen and Mohamadzadeh, 2013; Guerreiro et al., 2018). Studies conducted in recent years have suggested that the abnormal immune response of RA may be associated with the imbalance of gut microbiota (Xu et al., 2020; Brandl et al., 2021; Li and Wang, 2021). Changes in the normal gut microbiota impact mucosal immunity, affecting extraintestinal diseases, such as RA (Owen and Mohamadzadeh, 2013; Jiao et al., 2020). Patients with RA and other rheumatoid diseases have shown differences in the abundance of specific common gut microbiota compared to healthy controls (Maeda et al., 2016; Bodkhe et al., 2019). In an animal study, arthritis was alleviated in K/BxN mice under aseptic conditions (Teng et al., 2017). However, the germ-free mice developed more severe arthritis after being transplanted with the gut microbiota from collagen-induced arthritis (CIA) susceptible mice (Wu et al., 2010).

The imbalance of gut microbiota may lead to changes in metabolomic profiling in the human body which accounts for etiology of autoimmune diseases (Kim et al., 2017; Tsai et al., 2021; Yang and Cong, 2021). Metabolomics, the systematic study of the sets of metabolites in an organism, is an excellent tool to provide further insight or scientific basis into specific pathogeneses as well as the mechanism of actions of botanical medicines (Marchev et al., 2021; Oyenihi et al., 2021). In addition, ethnomedicine such as berberine can modulate the composition and metabolites of gut microbiota, and thus ameliorate the diseases (Cheng et al., 2021). Combined application of 16S rRNA sequencing and metabolomics, therefore, can help explain the mechanism of ethnic Medications through the metabolic interaction between the gut microbiota and the host (Cui et al., 2020a).

Twenty-five Wei’er tea pills (TFP), made of 25 Tibetan natural drugs (mostly botanical drugs), such as stem of *Senegalia catechu* (L.f.) P.J.H.Hurter and Mabb. (100 g), fruits of *Terminalia chebula* Retz. (100 g), fruits of *Terminalia billerica* (Gaertn.)Roxb. (125 g), fruits of *Phyllanthus emblica* L. (100 g), *Hymenidium hookeri* (C.B.Clarke) Pimenov and Kljuykov (50 g), rhizome of *Polygonatum sibiricum* Redouté (40 g), root of *Asparagus cochinchinensis* (Lour.) Merr. (40 g), root of *Oxybaphus himalaicus* Edgew. (25 g), fruits of *Tribulus terrestris* L. (30 g), resin of *Boswellia sacra* Flück. (*Boswellia carterii* Birdw.) (50 g), seeds of *Senna tora* (L.) Roxb. (50 g), seeds of *Abelmoschus manihot* (L.). Medik. (35 g), stem of *Tinospora sinensis* (Lour.) Merr. (100 g), fruits cluster of *Piper longum* L. (30 g), rhizome of *Acorus calamus* L. (acori calami rhizoma) (50 g), root of *Dolomiaea costus* (Falc.) Kasana and A.K.Pandey (aucklandiae radix) (50 g), *Oxytropis kansuensis* Bunge (40 g), fruits of *Rosa sweginzowii* Hemsl. and E.H.Wilson (50 g), flower of *Gentiana macrophylla* Pall. (30 g), root of *Aconitum pendulum* N.Busch (*Aconitum pendulum* Busch) (40 g), musk of *Moschus chrysogaster sifanicus* (30 g), shell of *Hyriopsis cumingii* (Lea) (25 g), horn of *Bubalus arnee f. bubalis* (25 g), is a traditional Tibetan medicine used to treat RA by relieving pain and inflammation and dispelling wind and numbness in clinical practice (Li, 2014). A recent case-control study revealed TFP’s remarkable curative effect and safety compared with conventional antirheumatic drugs (Zha, 2017). However, the mechanism of TFP relating to the treatment of RA has not been reported yet. In this study, we used the CIA rat model to evaluate the therapeutic effect of TFP on RA rats and explored the potential mechanism by a combination of 16S rRNA sequencing of gut microbiota and investigation on serum metabolites.

## Materials and Methods

### Materials

TFP was purchased from Tibet Ganlu Tibetan Medicine Co., Ltd. (Lhasa, China. Batch number: 20200502). Dexamethasone tablets, produced by South Land Pharmaceutical Co., Ltd., were obtained from Lhasa Jiming Pharmacy (Lhasa, China). The IL-1β, IL-4, IL-6, IL-10, IFN enzyme-linked immunosorbent assay (ELISA) kits were purchased from Wuhan Saipei Biotechnology Co., Ltd. (Wuhan, China). The immunohistochemistry kits were purchased from Solarbio Biotechnology Co., Ltd. (Beijing, China). The Bovine Type II Collagen-Solution was purchased from Amyjet Scientific Inc. (Wuhan, China).

### Animals

Male Sprague Dawley rats, aged 6–8 weeks and weighing 190–210 g, were selected and purchased from Beijing Huafukang Animal Co., Ltd. (Beijing, China) (Certificate No. SYXK (Jing) 2019–0022). They were conventionally raised for one week in a controlled environment (12-h light/dark cycle, temperature 21 ± 2°C, relative humidity of 45 ± 10%) and were given free access to food and water. All animal experiments were conducted under guidelines approved by the Animal Ethics Committee of Tibet University.

### Animal Experiment Procedures

#### CIA Rat Modeling

The collagen emulsion was prepared on a sterile table: first, the 0.05 M glacial acetic acid solution was prepared. Then, the Bovine Type II Collagen was weighed (protected from light) with an electronic balance and added into the glacial acetic acid solution to form a mixed solution with a concentration of 2 mg/ml. Next, the above-mixed solution was shaken using a laboratory shaker for 12 h at 4°C under dark conditions. Finally, an equal volume of complete Freund’s adjuvant was added into the shaken solution to form a water-insoluble mixture. The mixture was stored in an ice bath and kept away from light for later use.

After 1 week of acclimation, six rats were randomly selected to be used in the Control group, while the remaining 24 rats were used for modeling. Primary immunization of the models took place on day 0 as follows: after skin disinfection, the collagen emulsion was given at a dose of 0.2 ml/rat *via* subcutaneous injection at 2 cm from the base of the tail. Pressure was kept on the base of the tail for 30 s after administering the injection to prevent extravasation of the emulsion. Booster immunization of the models took place on day 7 as follows: the collagen emulsion was given at a dose of 0.1 ml/rat *via* subcutaneous injection at 2 cm from the base of the tail. Rats in the control group were injected with an equal volume of normal saline using an identical method.

#### Grouping and Drug Administration

On day 21 post-modeling, the twenty-four rat models were randomly divided into four groups evenly:

The Model group: each rat was given saline.
The Positive group: each rat was given dexamethasone for four consecutive weeks through intragastric administration, firstly with a dosage of 0.15 mg/kg/day for three consecutive days, then changed to 0.075 mg/kg/day.
The TFP low-dose (TFP-L) group: each rat was intragastrically administered 150 mg/kg/day of TFP for four consecutive weeks.
The TFP high-dose (TFP-H) group: each rat was given with 450 mg/kg/day of TFP for four consecutive weeks.

Another six normally fed rats were classified as the Control group.

The TFP (pills) were crushed and weighed, then the ground TFP was dispersed into water with a concentration of 150 mg/ml (stock suspension). The stock suspension was further diluted to concentrations that were needed for administration. The dexamethasone (tablets) was treated the same way as TFP to get a suspension of 150 mg/ml.

#### Sample Collection

Rats from all groups were anesthetized by pentobarbital sodium 24 h after the last TFP intervention. Then blood and fecal samples were collected. Blood was collected from the abdominal aorta and centrifuged at 3,000 r/min for 15 min at 4°C, then the supernatant was obtained and aliquoted for inflammatory factors assay and metabolomic test. Feces were collected under sterile conditions for 16S rRNA analysis. Both serum and stool samples were stored in a freezer at −80°C until analysis. In addition, the portion of the left hind foot at 1.5 cm above the ankle joints were removed for histopathological observation after all rats were euthanized.

### Pharmacodynamics Indexes Assay

#### Measurement of Basic Physiological Indices

The total duration of the animal experiment lasted 49 days. Rats were weighed every 5–7 days from the first day of the experiment. The bodyweight (in g) of the rats was recorded.

After the initial dose of the drug was administered, the anteroposterior diameter of the left ankle joint, as well as the thickness of the left sole, were measured and recorded (in cm) every 5–8 days using a vernier caliper.

#### Detection of Serum Inflammatory Factors

All rats were anesthetized on day 49 and serum was collected to measure the inflammatory factors in the ELISA kit to detect inflammatory factors, such as TNF-α, IL-1β, IL-4, IL-6, and IL-10.

#### Histopathological Observation

The abovementioned left ankle joints (see 2.3.3) were immediately fixed in the 10% formalin, and decalcified with 15% ethylenediaminetetraacetic acid (EDTA) for 30 days. After which, the same tissue was dehydrated with different concentrations of ethanol step-by-step, embedded in paraffin, sliced (using a Leica RM2125, Buffalo Grove, United States), and stained with hematoxylin and eosin (HE). Pathological features of the synovial tissue were observed under an optical microscope, especially tissue involving inflammatory cell infiltration and hyperplasia of the synovial tissue, pannus, and cartilage erosion.

#### Data Analysis

Measurement data of basic physiological indices and inflammatory factors were reported as mean ± standard deviation (SD). Statistical differences were tested between the experimental groups using SPSS 21.0 with one way Analysis of Variance, or a nonparametric test (Kruskal–Wallis test). *p* < 0.05 was considered statistically significant.

### Fecal 16S rRNA Sequencing, Data Processing and Analysis

After 4 weeks of medication, feces were collected under sterile conditions from the control, model, and TFP groups. The DNA in the feces was extracted and determined through agarose gel electrophoresis. Then, the DNA samples were diluted to 1 ng/μL. The 16S rRNA V4 region of the DNA samples were amplified by polymerase chain reaction (PCR) using specific primers (forward: GTGCCAGCMGCCGCGGTAA; reverse: GGACTACHVGGGTWTCTAAT). Four deoxynucleoside triphosphates (dNTP) were mixed 0.2 μL primers and 10 ng of template DNA to make up the amplification mixture of each sample. Subsequently, PCR products were quantified through 2% agarose gel electrophoresis and purified using the Qiagen Gel Extraction Kit (Qiagen, Germany). The sequencing library was completed using TruSeqRDNA PCR Sequencing Preparation Kit (Illumina, United States) and then sequenced on the NovaSeq6000 platform to generate paired-end reads.

Barcode and primer sequences were removed from the raw reads obtained from the above 16S rRNA sequencing, and reads from the same sample were merged using the flashv1.2.7 analysis tool (http://ccb.jhu.edu/software/FLASH/) to obtain the raw marks. Then the raw marks were filtered using the software Qiime (V1.9.1, http://qiime.org/scripts/split_libraries_fastq.html) quality Control process to obtain clean tags. Furthermore, chimeric sequences were detected and removed from the clean data using the UCHIME algorithm to obtain valid data. Afterwards, the software Uparse (Uparse v7.0.1001, http://www.drive5.com/uparse/) was used to assign valid sequences with a similarity of ≥97% to the same OTU (Operational Taxonomic Units) and the representative sequence with the highest frequency was filtered for each OUT. Subsequently, species annotation analysis was carried out through the Silva database (http://www.arb-silva.de/). As a result, the taxonomic information was obtained and the community composition of each sample was counted at each taxonomic level, such as phylum, genus and species. After that, the indexes reflecting alpha or beta diversity were calculated by the software Qiime V1.9.1, such as Observed-species, Chao1, Shannon, Unifrac distance, et al. Moreover, the gene family abundance was predicted using a phylogenetic investigation of communities by reconstruction of unobserved states (PICRUSt).

The quantitative data of alpha diversity, such as the observed_species, chao1 index and Shannon, were analyzed and visualized using SPSS 21.0. Data of beta diversity was analyzed and visualized in the statistical software R (Version 3.4.3). Results with *p* < 0.05 were considered statistically significant.

### Untargeted Metabolomics Test

#### Sample Preparation for Metabolomics Analysis

The samples (100 μL) were placed in the EP tubes and resuspended with prechilled 80% methanol by well vortex. The mixtures were then incubated on ice for 5 min and centrifuged at 15,000 rpm, 4°C for 20 min. Afterwards, the supernatant was diluted to final concentration containing 53% methanol by LC-MS grade water. Subsequently, the treated samples were transferred to a fresh Eppendorf tube and centrifuged at 15,000 rpm, 4°C for 20 min. Finally, the supernatant was injected into the LC-MS/MS system. Meanwhile, quality control (QC) sample was prepared for analysis by mixing equal amount of the final supernatant of each sample.

#### LC-MS Conditions

Metabolomic profiling was analyzed using UHPLC-MS/MS. The UPLC was conducted on Dionex U3000 UPLC system (ThermoFisher, Germany) coupled with ACQUITY UPLC BEH C18 column (100 × 2.1 mm, 1.7 μm, Waters Corporation) at 45°C. The mobile phase was comprised of eluent A (0.1% formic acid in water, v/v) and eluent B (methanol) and the gradient program was: 2% B, 1.5 min; 2–85% B, 3 min; 100% B, 10 min; 100–2% B, 10.1 min; 2% B, 12 min.The MS was carried out on an Orbitrap Q ExactiveTM HF mass spectrometer (Thermo Fisher, Germany). The electrospray ionization source (ESI) in both positive and negative ion modes was used with spray voltage of 3.5 kV, capillary temperature of 320°C, sheath gas flow rate of 35 arb and aux gas flow rate of 10 arb, S-lens RF level of 60, Aux gas heater temperature of 350°C.

#### Data Processing and Analysis

The raw data files generated by UHPLC-MS/MS were processed using the Compound Discoverer 3.1 software (CD3.1, ThermoFisher) to perform peak alignment, peak picking, and quantitation for each metabolite. The main parameters were set as follows: retention time tolerance, 0.2 min; actual mass tolerance, 5 ppm; signal intensity tolerance, 30%; signal/noise ratio, three; and minimum intensity, et al. After that, peak intensities were normalized to the total spectral intensity. The normalized data was used to predict the molecular formula based on additive ions, molecular ion peaks and fragment ions. And then peaks were matched with the mzCloud (https://www.mzcloud.org/), mzVault and MassList database to obtain the accurate qualitative and relative quantitative results. Statistical analyses were performed using the statistical software R (R version R-3.4.3), Python (Python 2.7.6 version) and CentOS (CentOS release 6.6).

The processed data were analyzed using the method described by Cui et al. (2020b). Principal components analysis (PCA) and Partial least squares discriminant analysis (PLS-DA) were performed at metaX (a flexible and comprehensive software for processing metabolomics data) (Wen et al., 2017). Univariate analysis (*t*-test) was used to calculate the statistical significance (*p*-value). Metabolites with variable importance in projection (VIP) > 1, fold change (FC) value > 1.2 or <0.8 and *p* < 0.05 between the Control and Model groups or between the TFP and model groups are considered as differential metabolites. The False Discovery Rate (FDR) was also calculated using the statistical software R and the differential metabolites with the reported values of FDR less than 0.05 were supposed to be statistically significant. In addition, pathway analysis was conducted using the MetaboAnalyst 5.0 software (https://www.metaboanalyst.ca/).

### Correlation Analysis of the Gut Microbiota and Metabolites

The spearman correlations were analyzed between significant differential metabolites and altered species of gut bacteria and visualized using the software R. P-value < 0.05 was considered as statistically significant. Furthermore, pathways were intersected between differential metabolites and altered species of gut bacteria.

### Chemical Composition Characterization

#### Sample Preparation

TFP was weighed accurately and extracted using ultrasonic assisted extraction with methanol. The extract was then concentrated and re-dissolved by methanol to prepare a sample solution of 0.7 g/ml. The sample solution was filtered with 0.22 μM microporous membrane for high performance liquid chromatograph (HPLC) analysis.

Standard substances were dissolved in methanol separately to prepare standard solutions, which were also filtered with 0.22 μM microporous membrane for HPLC test.

#### HPLC Conditions

The HPLC was performed on Agilent 1200 HPLC system (Agilent Technologies Inc., United States) coupled with Agilent HC-C_18_ column (250 × 4.6 mm, 5 μm) at 35°C. The mobile phase was composed of eluent A (0.03% acetic acid in water, v/v) and eluent B (methanol: acetonitrile, 11:9, v/v), with a gradient set as following: 5–15% B, 20 min; 15–18% B, 35 min; 18–55% B, 60 min; 55–78% B, 80 min. The flow rate was 0.9 ml/min.

#### Data Analysis

Retention time of a sample peak was compared with peaks of standard substances and the retention time tolerance was set at 0.3 min. Spectral characteristics of the sample peaks were also compared with those of standard substances for further confirmation. Then the Chromatograms of samples and standard substances were imported into the Similarity evaluation system A of chromatographic fingerprint of traditional Chinese medicine (version Year-2012) (Zou and Yan, 2018) for visualization.

## Results

### Effect of TFP on the Body Weight, Sole Thickness, and Joint Diameter of CIA Models Rats

**FIGURE 1:**
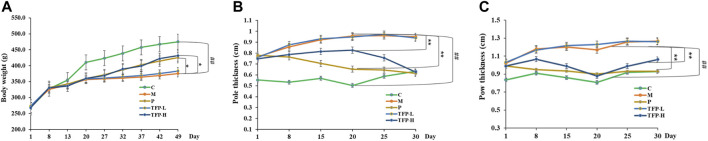
**(A)**: Effect of TFP on the bodyweight; **(B)**: Effect of TFP on the ankle joint diameter; **(C)**: Effect of TFP on the sole thickness. ##: Significantly different compared with the Control group (*p* < 0.01); *: Significantly different compared with the Model group, **: (*p* < 0.01), *: (*p* < 0.05).

After 1 week of modeling, we observed the following changes in the studied rats (with the exception of the control group): slowed weight gain, loss of appetite, dried hair, and joint redness/swellin. After drug administration, body weight was reduced in the model group compared with the control group (*p* < 0.01); weight gain became significantly faster in the Positive and TFP-H groups compared with the Model group (*p* < 0.05). No significant difference was noted in the bodyweight changes when comparing the TFP-L group and the Model group (Figure 1A).

The ankle joint diameter of the left foot and the thickness of the left sole varied significantly between the Model group and Control group (*p* < 0.01). The two indicators were improved significantly in the Positive and TFP-H groups (*p* < 0.01), but did not show distinct differences in the TFP-L group compared with the Model group (Figures 1B,C).

### TFP Therapy Improved the Inflammatory Response of CIA Rats and the Pathological Phenomena of Their Ankle Joints

Compared with the Control group, the expression level of TNF-α, IL-1β, and IL-6 increased significantly (*p* < 0.05, 0.01, and 0.01, respectively), and the expression level of IL-4 and IL-10 decreased significantly (both *p* < 0.01) in the Model group and TFP-L group. Compared with the Model group, the expression level of TNF-α and IL-6 decreased (*p* < 0.05 and 0.01, respectively), and the expression level of IL-4 and IL-10 increased (both *p* < 0.01) in the Positive and TFP-H groups. No significant difference was noted between the TFP-L group and Model group (Table 1).

**TABLE 1:** Changes of inflammatory factors after TFP therapy.

Group	TNF-α	IL-1β	IL-4	IL-6	IL-10
Control	1063.34 ± 27.48	30.59 ± 5.48	674.4 ± 67,72	44.4 ± 1.93	555.42 ± 60.23
Model	1220.97 ± 75.85^#^	72.36 ± 25.65^##^	351.31 ± 24.22^##^	100.29 ± 14.12^##^	365.52 ± 34.27^##^
Positive	1056.03 ± 45.35^∗^	33.88 ± 3.27 ^∗^	598.53 ± 50.27 ^∗∗^	55.42 ± 7.26 ^∗∗^	513.74 ± 38.38^∗∗^
TFP-L	1182.79 ± 98.5^#^	65.34 ± 14.29^##^	378.36 ± 20.12^##^	92.89 ± 9.69^##^	390.94 ± 22.94^##^
TFP-H	1061.07 ± 27.67^∗^	41.3 ± 7.88	625.64 ± 89.19 ^∗∗^	60.74 ± 8.66 ^∗∗^	495.38 ± 60.21^∗∗^

Note: Data were reported as mean ± SD (pg/ml). #: compared with the Control group (*p* < 0.01 as ##, *p* < 0.05 as #); *: compared with the Model group (*p* < 0.01 as **, *p* < 0.05 as *).

Compared with the Model group, no significant improvement was found in the bodyweight, joint swelling, or expression levels of inflammatory factors in the TFP-L group. Therefore, the TFP-L group was excluded, and the TFP-H group was named as TFP group in subsequent tests.

**FIGURE 2:**
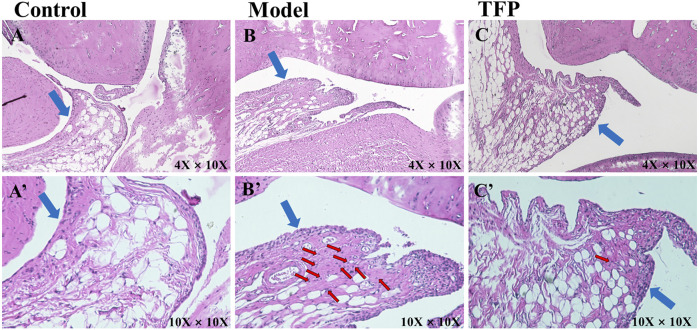
H and E staining of ankle joint tissue (×10 objective lens on a microscope); the blue arrows point at synovium and the red arrows point at inflammatory cell infiltration. **(A)**/**(A’)**: Joint tissue slice of rats in Control group (4X/10X eyepiece); **(B)**/**(B’)**: Joint tissue slice of rats in Model group (4X/10X eyepiece); **(C)**/**(C’)**: Joint tissue slice of rats in TFP group (4X/10X eyepiece).

HE staining was then performed to the ankle joint slice of the left hind foot. The results are shown in Figure 2. Rats in the Control group were observed to have structurally complete articular cartilage, evenly distributed chondrocytes, and no pathological changes in the joints (see Figure 2A). Massive inflammatory cell infiltration, synovial hyperplasia, fibrous hyperplasia, and other pathological changes were found in the Model group rats (see Figure 2B). Compared with the Model group, the inflammatory response of the ankle joint in the TFP group was significantly improved (see Figure 2C).

### Regulation of TFP on the Gut Microbiota of CIA Rats

**FIGURE 3:**
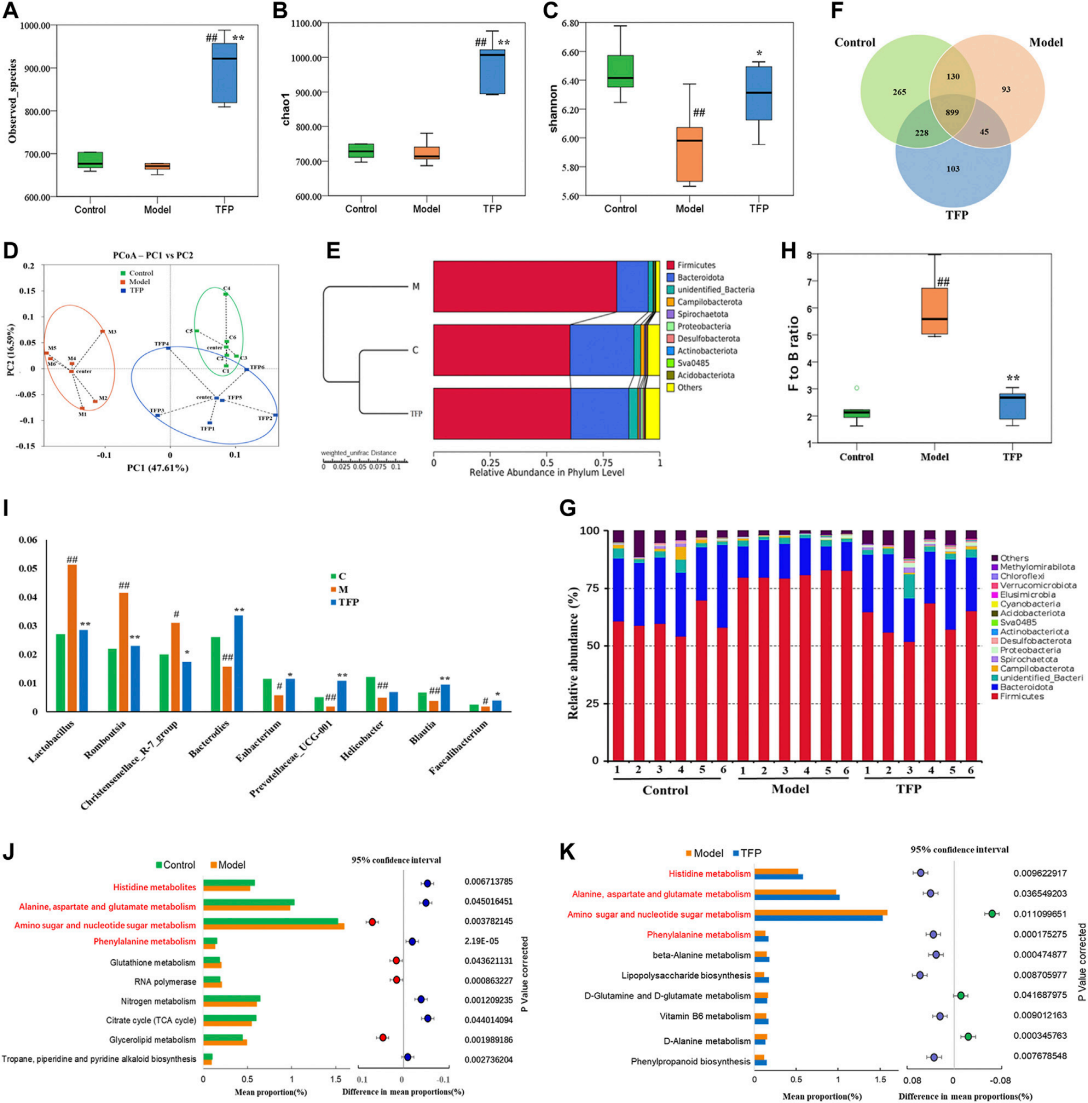
TFP therapy affected the gut microbiota of RA model rats. **(A,B)** Chao1 index and Observed_species for each group, where TFP group significantly increased. No significant difference was noted between the Model group and the Control group. **(C)** The Shannon index was significantly lower in the Model group than in the Control group. No significant difference was noted between the TFP group and the Control group. **(D)** In terms of PCoA, the TFP group was closer to the Control group. **(E)** The hierarchical cluster tree showed that the TFP group had a more similar Beta diversity with the Control group. **(F)** Different numbers of OTUs are shown in the Venn diagram. **(G,H)** At the phylum level, TFP lowered the Firmicutes to Bacteroidetes (F to B) ratio of RA model rats. **(I)** TFP affected the relative abundance of Bacteroides, Eubacterium, Prevotellaceae_UCG-001, Blautia, Faecalibacterium, Lactobacillus, Romboutsia, and Christensenellace_R-7_group at the genus level in RA model rats. **(J,K)** Based on 16S rRNA sequencing data, the PICRUSt analysis was used to predict different metabolic pathways of TFP in RA. Control, Model, and TFP groups (*n* = 6 per group).

A total of 1,068,736 Effective Tags and 1,763 OTUs were obtained from 18 samples. The 16S rRNA sequencing of gut microbiota from the rat cecum of the three groups showed gut microbiota changes in RA rat models before and after TFP therapy. Both the observed_species and Chao1 indexes demonstrated no significant difference in the number of gut microbial species between the Model and Control groups. In contrast, the number of gut microbial species increased significantly in rats receiving the TFP therapy (*p* < 0.01 *vs*. Control and Model group) (Figures 3A,B). The Shannon index showed that the evenness of gut microbiota in the Model group was significantly changed compared with that of the Control group (*p* < 0.01), and it returned to a normal level after TFP therapy (*p* < 0.05 *vs*. Model group) (Figure 3C).

The principal co-ordinates analysis (PCoA) based on the Weighted UniFrac algorithm suggested that the control, model, and TFP groups differed significantly in the gut microbiota/microbial colony. The gut microbiota of RA rats mainly aggregated on the left of axis 0 before treatment but on the right of axis 0 after treatment. The distance between the TFP and control groups was closer than the distance between the TFP and Model groups (Figure 3D). The hierarchical cluster tree also revealed a closer distance between the TFP and Control groups (Figure 3E).

We obtained 1,763 OTUs by analyzing 16s rRNA of the gut microbiota. The Venn diagram showed 899 overlapping OTUs in all groups, and there were 103 specific OTUs in the Model group, 93 in the Control group, and 265 in the TFP group (Figure 3F).

At the phylum level, Firmicutes and Bacteroidetes were the most abundant phyla in all microbial colonies (Figure 3G). The Model group had a significantly higher abundance of Firmicutes (*p* < 0.01) but a significantly lower abundance of Bacteroidetes (*p* < 0.01) than the control. No statistical difference was found between the TFP and control groups in the abundance of Firmicutes and Bacteroidetes. The differences in *the Firmicutes to Bacteroidetes* (F to B) ratio between the groups are shown in Figure 3H. In addition, abundance of Desulfobacterota in the Model group (*p* < 0.05) was significantly reduced compared to that in the control group, and was increased in the TFP group compared with the model group (*p* < 0.05).

At the genus level, the relative abundance of *Bacteroides*, Eubacterium, *Prevotellaceae_UCG-001*, *Helicobacter*, Blautia, and Faecalibacterium was significantly reduced (*p* < 0.01, 0.05, 0.01, 0.01, 0.01, and 0.05, respectively, Figure 3I), and the relative abundance of *Lactobacillus*, Romboutsia, and Christensenellace_R-7_group was significantly increased (*p* < 0.01, 0.01, and 0.05, respectively, Figure 3I) in the Model group when compared with the control group. The relative abundance *of Bacteroides, Eubacterium, Prevotellaceae_UCG-001, Blautia and Faecalibacterium was* increased (*p* < 0.01, 0.01, and 0.05, respectively, Figure 2I) and the relative abundance of *Lactobacillus*, Romboutsia, Christensenellace_R-7_group was reduced (*p* < 0.01, 0.01, and 0.05, respectively, Figure 3I) in the TFP group when compared with the model group.

The functions of different gut microbiota at the genus level were predicted using the PICRUSt. The top 10 metabolic pathways with the highest proportion and *p* < 0.05 are shown in Figure 3J (comparison between the control group and the model group) and Figure 3K (comparison between the model and TFP group). Compared with the control group, the abundances of amino sugar and nucleotide sugar metabolism, glycerolipid metabolism, RNA polymerase and glutathione metabolism, increased, while abundances of others decreased; such as histidine metabolism, phenylalanine metabolism, as well as alanine, aspartate and glutamate metabolism (Figure 3J). Compared with the model group, the TFP group had a lower abundance of amino sugar and nucleotide sugar metabolism and a higher abundance of histidine metabolism, phenylalanine metabolism, and alanine, aspartate and glutamate metabolism (Figure 3K).

### Effect of TFP on Serum Metabolism in CIA Rats

**FIGURE 4:**
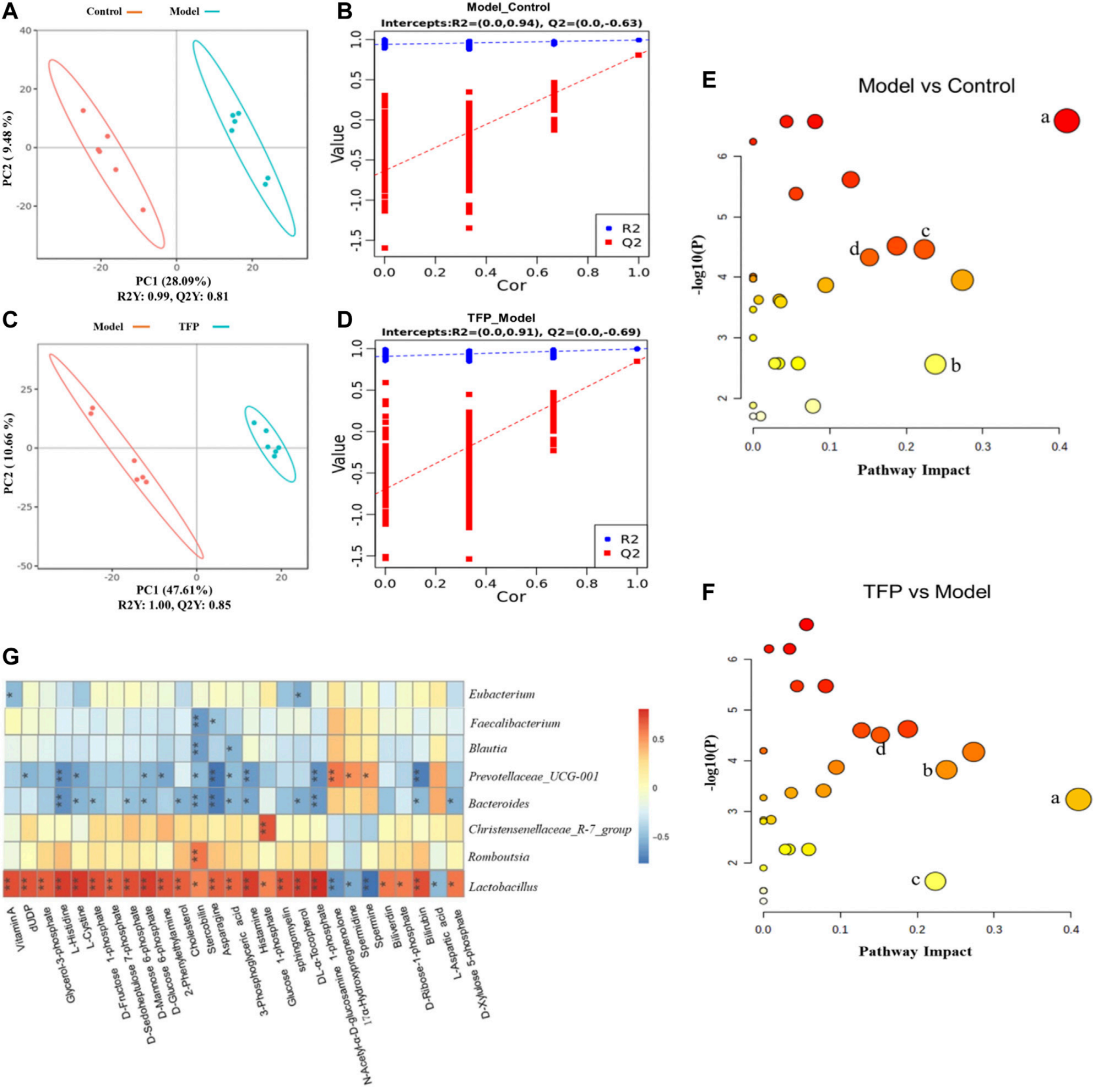
TFP therapy regulated the metabolites in serum of CIA rats and the correlations with gut microbiota. **(A, C)**: PCoA scores of OPLS-DA between Control/Model groups and between Model/TFP groups. **(B,D)**: OPLS-DA loading coefficients between Control/Model groups and between Model/TFP groups. **(E,F)**: Analysis on pathways of the 27 differential metabolites. Commo pathways between those obtained from PICRUSt of 16S rRNA sequencing and those related to untargeted metabolomics (*p* < 0.05, Impact >0.1) were remarked as a: Histidine metabolism; b: Phenylalanine metabolism; c: Alanine, aspartate and glutamate metabolism; d: Amino sugar and nucleotide sugar metabolism **(G)**: Spearman’s analysis between the 27 differential metabolites and the gut microbiota modulated by TFP at genus level.

Changes of metabolites in serum were investigated using untargeted metabolomics. The Principal Component Analysis (PCA) model showed the significant group separation between the Control and Model groups and between the Model and TFP groups (Figures 4A,C). The orthogonal projections to latent structures (OPLS-DA) model also showed a significant difference in metabolomics data between the control and model groups and between the model and TFP groups. Overfitting in the OPLS-DA model was controlled by seven rounds of cross-validation and 200 repetitions (RPT) based on R2 and Q2 values (Figures 4B,D). 27 common differential metabolites between control/model and model/TFP groups were identified and FDR calculation confirmed the statistical significances. The significant differential metabolites are: Vitamin A, dUDP, Glycerol-3-phosphate, L-Histidine, L-Cystine, D-Fructose 1-phosphate, D-Sedoheptulose 7-phosphate, D-Mannose 6-phosphate, D-Glucose 6-phosphate, 2-Phenylethylamine, Cholesterol, Stercobilin, Asparagine, 3-Phosphoglyceric acid, Histamine, Glucose 1-phosphate, sphingomyelin, DL-α-Tocopherol, N-Acetyl-α-D-glucosamine 1-phosphate, Biliverdin, D-Ribose-1-phosphate, Bilirubin, and D-Xylulose 5-phosphate (Table 2). Pathway analysis resulted in a total of 29 metabolic pathways as shown in Figures 4E,F, and they were further analyzed below.

**TABLE 2:** The differential metabolites in serum after TFP therapy.

NO	RT[min]	m/z	Formula	Name	VIP			FC		Trend	Pathway
					M vs C	TFP vs M	M vs C	TFP vs M	M vs C	TFP vs M	
1	15.623	285.222	C_20_H_30_O	Vitamin A	1.67	1.10	2.59	0.57	↑^##^	↓*	−
2	1.162	387.001	C_9_H_14_N_2_O_11_P_2_	dUDP	2.03	1.83	4.59	0.27	↑^##^	↓**	−
3	1.194	171.006	C_3_H_9_O_6_P	Glycerol-3-phosphate	2.46	1.43	5.53	0.36	↑^##^	↓**	−
4	1.934	154.062	C_6_H_9_N_3_O_2_	L-Histidine	1.96	1.94	1.79	0.58	↑^##^	↓**	a
5	1.204	239.017	C_6_H_12_N_2_O_4_S_2_	L-Cystine	1.74	1.30	3.08	0.49	↑^##^	↓*	−
6	1.185	221.067	C_6_H_13_O_9_P	D-Fructose 1-phosphate	2.16	1.81	2.84	0.40	↑^##^	↓**	d
7	1.192	253.057	C_7_H_15_O_10_P	D-Sedoheptulose 7-phosphate	1.98	1.67	4.01	0.31	↑^##^	↓**	−
8	1.184	259.022	C_6_H_13_O_9_P	D-Mannose 6-phosphate	2.09	1.80	3.04	0.36	↑^##^	↓**	d
9	1.182	223.046	C_6_H_13_O_9_P	D-Glucose 6-phosphate	2.10	1.96	2.60	0.40	↑^##^	↓**	d
10	5.933	122.097	C_8_H_11_N	2-Phenylethylamine	1.33	2.14	3.33	0.11	↑^##^	↓**	b
11	15.175	387.362	C_27_H_46_O	Cholesterol	1.63	1.22	1.83	0.60	↑^##^	↓*	−
12	10.184	595.350	C_33_H_46_N_4_O_6_	Stercobilin	1.20	1.58	1.99	0.45	↑^#^	↓*	−
13	1.225	131.046	C_4_H_8_N_2_O_3_	Asparagine	1.67	1.70	1.69	0.60	↑^##^	↓**	a,c
14	1.216	192.988	C_3_H_8_NaO_6_P	3-Phosphoglyceric acid	1.75	1.82	3.28	0.30	↑^##^	↓**	−
15	1.069	112.087	C_5_H_9_N_3_	Histamine	1.78	1.49	2.15	0.52	↑^##^	↓**	a
16	1.489	261.037	C_6_H_13_O_9_P	Glucose 1-phosphate	1.09	1.84	2.38	0.20	↑#	↓**	d
17	15.452	759.567	C_39_H_77_N_2_O_6_P	Sphingomyelin	1.92	1.22	2.01	0.64	↑##	↓*	−
18	15.111	429.374	C_29_H_50_O_2_	DL-α-Tocopherol	1.99	1.25	2.25	0.59	↑##	↓**	−
19	1.19	262.093	C_8_H_16_NO_9_P	N-Acety-D-glucosamine1-phosphate	2.08	1.67	2.48	0.50	↑##	↓**	−
20	13.163	331.228	C_21_H_32_O_3_	17-Hydroxypregnenolone	1.53	1.54	0.39	2.31	↓##	↑**	−
21	1.092	129.139	C_7_H_19_N_3_	Spermidine	1.90	1.77	0.40	2.28	↓##	↑**	−
22	1.109	203.223	C_10_H_26_N_4_	Spermine	1.82	1.06	0.61	1.63	↓#	↑*	−
23	15.612	583.255	C_33_H_34_N_4_O_6_	Biliverdin	1.34	0.47	2.30	0.69	↑#	↓	−
24	1.207	191.056	C_5_H_11_O_8_P	D-Ribose-1-phosphate	1.71	1.48	2.39	0.54	↑##	↓**	−
25	12.596	583.257	C_33_H_36_N_4_O_6_	Bilirubin	1.54	1.15	1.57	0.71	↑##	↓**	−
26	1.195	132.030	C_4_H_7_NO_4_	L-Aspartic acid	1.19	1.53	0.68	1.43	↓#	↑*	c
27	1.192	229.012	C_5_H_11_O_8_P	D-Xylulose 5-phosphate	1.62	1.70	1.45	0.49	↑##	↓**	−

Note: Control, Model, and TFP, groups (*n* = 6 per group). #, *p* < 0.05 compared with the Control group; ##, *p* < 0.01 compared with the Control group; *, *p* < 0.05 compared with the Model group; **, *p* < 0.01 compared with the Model group; ↑content increased; ↓, content decreased; vs, versus; C: control group; M: model group; TFP: TFP, group. (a) Histidine metabolism. (b) Phenylalanine metabolism (c) Alanine, aspartate and glutamate metabolism. (d) Amino sugar and nucleotide sugar metabolism.

### Correlation Analysis Between Untargeted Metabolomics and Gut Microbiota

Common pathways were investigated between those obtained from PICRUSt of 16S rRNA sequencing and those related to the differential metabolomics (*p* < 0.05, Impact >0.1), which resulted in four pathways: Histidine metabolism, Phenylalanine metabolism, Alanine, aspartate and glutamate metabolism, as well as amino sugar and nucleotide sugar metabolism (remarked in red in Figures 3J,K and noted with ‘a’, ‘b’, ‘c’, ‘d’ in Figures 4E,F).

Spearman’s analysis was conducted between the 27 differential metabolites and the gut microbiota modulated by TFP at genus level (Figure 4G). *Lactobacillus* and Christensenellaceae_R-7_group had significant positive correlations with the abnormal changes of serum metabolites in RA rat models. In contrast, *Bacteroides* and *Prevotellaceae_UCG-001* showed negative correlations with those changes in CIA rat models. Notably, *Lactobacillus* exhibited correlations with all the metabolites.

### Chemical Composition of TFP

Supplementary Figure S1 and Figure 2 exhibited chemical composition profile of TFP methanol extract obtained through HPLC. Seven major ingredients were identified: ferulic acid, gallic acid, catechin hydrate, gentiopicroside, ellagic acid, piperine, and dehydrocostus lactone. Their respective structures are showed in Supplementary Material S3.

## Discussion

The complexity of RA calls for multi-target therapeutics (He et al., 2016). Studies have shown that ethnomedicine has a unique effect on the treatment of RA (Rather et al., 2013; Xiao et al., 2018; Li et al., 2021). The traditional Tibetan medicine, TFP, has a significant clinical effect in the treatment of RA, but its mechanism of action has not yet been studied.

The collagen-induced RA model is a well established and widely used animal model used to explore the mechanism of RA as well as the pharmacological effects of relevant trial drugs; this is because the comprising tissue the model is made up of produces similar features to human clinical RA in histopathological and immunological terms (Bessis et al., 2017; Lu et al., 2018). In this study, RA models were established in CIA rats to evaluate the therapeutic effect of TFP on RA. CIA model rats treated with TFP showed significant improvement in weight gain, pathological phenomena in joints, as well as decreased serum levels of TNF-α, IL-6 and increased level of IL-4 and IL-10.

Both TNF-α and IL-6 are important pro-inflammatory cytokines in the progression of RA (Tanaka et al., 2014; McInnes et al., 2016; Mateen et al., 2016). For example, TNF-α can mediate the activation of the NF-kB pathway, promote RA synovitis, and promote pannus formation (Medoff et al., 2009; Piga et al., 2014). IL-6 promotes the differentiation of Th17 cells and inhibits the differentiation of Treg cells (Tanaka et al., 2014); while IL-6 receptor antagonist reduces the inflammatory disease activity of RA (McInnes et al., 2015). IL-4 and IL-10, as anti-inflammatory cytokines, can limit the inflammatory process of RA (Leung et al., 2002; Finnegan et al., 2003; Henningsson et al., 2012; Lin et al., 2015). Studies have indicated arthritis is aggravated in CIA rats receiving anti-IL-10 antibodies (Finnegan et al., 2003), and the use of IL-10 significantly eased the collagen-induced joint swelling, infiltration, cytokine synthesis, cartilage deformity, and necrosis (Henningsson et al., 2012). IL-4 are used to treat various autoimmune models *in vivo*(Haikal et al., 2019); itregulates the polarization and activity of macrophages by inhibiting the Th1-mediated pro-inflammatory effect, thereby enhancing the Th2-mediated anti-inflammatory effect (Lin et al., 2015).

As a chronic autoimmune disease, the inflammation of RA cannot resolve spontaneously; therefore, the homeostasis of inflammation regulation, namely inhibiting the development of inflammation and promoting the inflammation resolution, may be a new strategy for the treatment of RA (Chen Z. et al., 2019). This study showed that TFP blocked the development of inflammation in RA rats not only by inhibiting the expression of inflammatory cytokines (TNF-α, IL-6), but also by increasing the expression of anti-inflammatory cytokines (IL-4 and IL-10). Such two-way regulation may be an advantage of TFP in treating RA, although the mechanism behind it needs further investigation.

Furthermore, we found that CIA model rats that had reduced diversities of gut microbiota were restored by TFP therapy. This is consistent with findings from other researchers, which showed that decreased diversity of gut microbiota in RA patients or RA model animals can be restored to their normal levels after treatment (Xiao et al., 2018; Li et al., 2021; Nayak et al., 2021). It should be noted that we observed a remarkable increase in gut microbiota organism abundance in TFP groups, which might be an indicator of improving pathologies. A recent cohort study based on meta-genome shotgun sequencing showed that the species richness of intestinal flora in RA patients with clinical improvement was higher than those without improvement in disease activity within 6 ∼ 12 months (Gupta et al., 2021).

We found RA rats had an increased abundance of Firmicutes and a decreased abundance of Bacteroidetes compared with normal rats, which is in line with findings from a previously published report (Rogier et al., 2017). Moreover, such changes were reversed and restored to the levels in normal rats after TFP therapy. Firmicutes and Bacteroidetes are the main phyla of gut microbiota in humans and rats (Jandhyala et al., 2015; Li et al., 2021). Many of their subordinate microorganisms participate in digesting the complex polysaccharide in their host’s food and produce short-chain fatty acids (SCFAs), the primary energy source of colonic mucosa, as well as essential regulators in host cell gene expression, inflammation, differentiation, and apoptosis (Hamer et al., 2008; Rosser et al., 2020).

At the genus level, TFP up-regulates the decreased abundance of *Bacteroides*, Eubacterium, *Prevotellaceae_UCG-001*, Blautia and Faecalibacterium, and downregulates the increased relative abundance of *Lactobacillus*, Romboutsia, Christensenellaceae_R-7_group in RA rats. In addition, 27 differential metabolites were identified and their relationship with the above eight genera of gut microbiota were analyzed. Four common metabolic pathways were found to be related to TFPs’ therapeutic effects by regulating metabolic pathways and mediating gut microbiota, including the metabolism of: Histidine, Phenylalanine, Alanine/Aspartate/Glutamate, as well as Amino- and nucleotide sugars.

**FIGURE 5:**
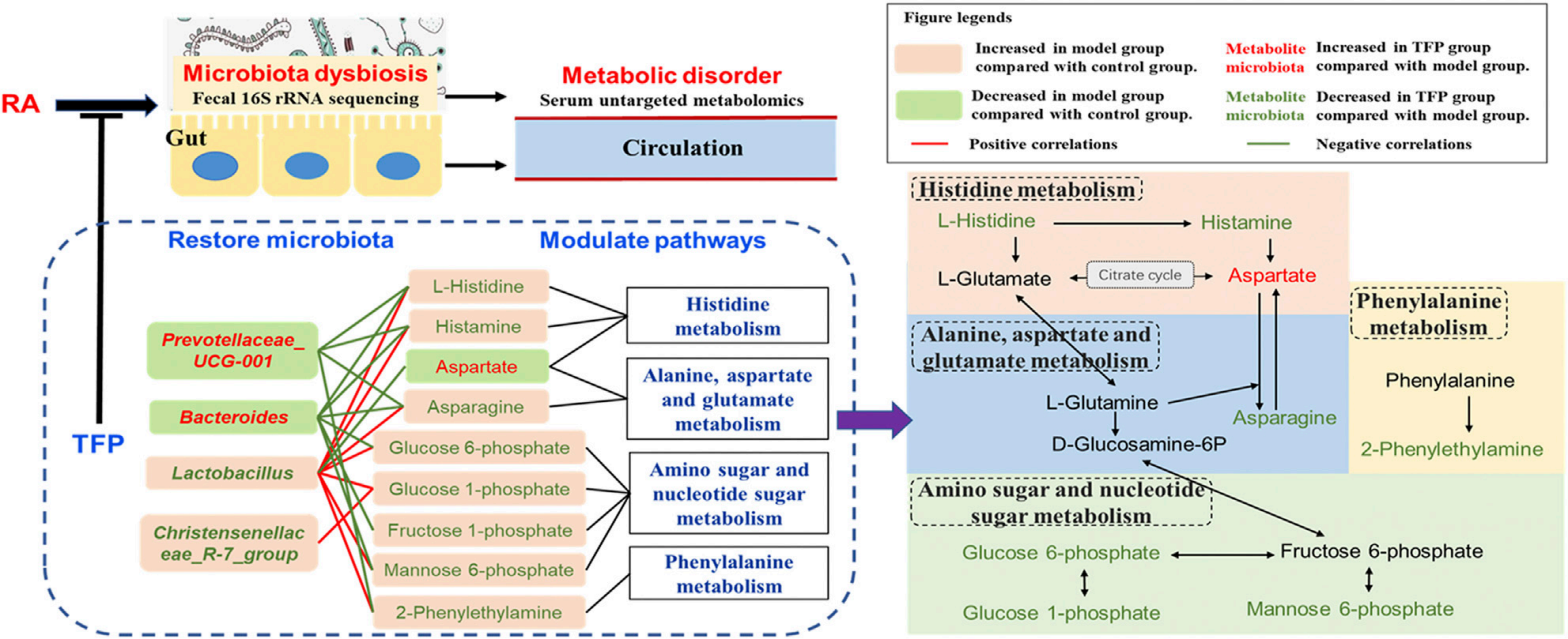
Schematic diagram of TFP’s mechanism based on modulation of gut Microbiota and metabolic pathways.

According to our findings, the KEGG pathway map and numerous literatures, we propose TFP’s working mechanisms by illustrating relationships of metabolites, pathways and gut microbiota as shown in Figure 5. TFP affects the abundances *of Lactobacillus, Christensenellace_R-7_group, Bacteroides, and* Prevotellaceae*_UCG-001,* and then influences metabolic pathways in CIA rats; the two processes are mediated by several key metabolites bound up with the above intestinal bacteria.

*Lactobacillus* are the most abundant flora genus in our study, in line with a previous research (Li et al., 2021). Numerous researches have reported significant increased gut *Lactobacillus* in RA patients (Picchianti-Diamanti et al., 2018), especially in active RA patients (Zhang et al., 2015; Qiao et al., 2020). This was as also noted in animals susceptible to collagen-induced arthritis (Liu et al., 2016; Qiao et al., 2020). On the other hand, some species of *Lactobacillus* such as *Lactobacillus casei* is widely accepted as probiotic and has potential to be used to ameliorate RA (Cannarella et al., 2021). Different species of *Lactobacillus* may play divergent roles. An increased abundance of *Christensenellaceae_R-7_group* was reported in mice feaces of mice treated with high fat, but it dropped after administering medication (Gong et al., 2020).

*Bacteroides*, members of Bacteroidetes, are SCFAs producing bacteria. Kaori Kitamura er al revealed a protective effect of *Bacteroides* on the inflammatory changes of RA mice (Kitamura et al., 2021). Jin ZL et al. reported a significant decline of *Bacteroides* in the gut of RA rats, but the abundance of *Bacteroides* were elevated after treating with probiotics (Jin and Chen, 2020). Prevotellaceae*_UCG-001* has been found significantly decreased in colitis model mice and could have a protective effect on the intestinal mucosal barrier function (Hu et al., 2020).

### Histidine Metabolism

Histamine, mediated by the activation of its four receptors (H1R–H4R), exhibits extensive effects related to visceral fat mass. It is known to function as a human neurotransmitter, a modulator of inflammatory and immune responses, and a key mediator of several allergic pathways and autoimmune pathogeneses (Mohammad et al., 2009). Innate and adaptive immune cells throughout the body can produce histamine by decarboxylation of the amino acid L-histidine by the enzyme histidine decarboxylase (HDC) (Jutel et al., 2009). Many bacteria are able to synthesize and secrete histamine as well and histamine has been considered as another central signal molecule that mediates bacteria–host interactions (Krell et al., 2021). For example, the administration of the histamine-secreting *Lactobacillus rhamnosus* had an anti-inflammatory effect by reducing the secretion of various interleukines and tumor necrosis factor *α* (Frei et al., 2013). However, another *Lactobacillus* species, L. saerimneri, which is able to secrete approximately 100-fold more histamine than L. rhamnosus, resulted in animal weight loss and signs of deteriorating health (Ferstl et al., 2014). Chen H et al. reported that *M. morganii* and L. reuteri strains generate histamine *in vitro* as well as *in vivo*, and that L-histidine significantly increases histamine production (Chen H. et al., 2019).

In our study, L-histidine and histamine was seen to have a positive correlation with *Lactobacillus* and a negative correlation with *Bacteroides* and *Prevotellaceae_UCG-001*. *Lactobacillus* revealed an unfavorable association with TFP’s regulation of the pathway, whereas *Bacteroides* and *Prevotellaceae_UCG-001* had beneficial effects. It is likely that these bacteria have an impact on the utilization of exogenous L-histidine/histamine and (or) production of histamine by the host.

### Phenylalanine Metabolism

Phenylalanine metabolism disorder with altered level of Phenylethylamine (PEA) has been suggested in RA models and RA patients by numerous studies (Xu et al., 2017; Liu et al., 2019; Tang et al., 2021). PEA is produced *via* decarboxylation of phenylalanine by the aromatic L-amino acid decarboxylase. Some gut bacteria such as *B. theta* could produce phenylalanine, a substrate for other intestinal bacteria stains to generate PEA; this metabolic exchange among gut bacteria has contributed to *in vivo* production of PEA and resulted in its increased level in host serum. (Chen H. et al., 2019). In addition, decreased PEA level in blood has been reported to be associated with improvement of osteoarthritis in patients (Smith-Ryan et al., 2020). Our study suggests that the elevated level of 2-Phenylethylamine (PEA) in RA rat serum was restored to normal by TFP through phenylalanine metabolism with the participation of *Lactobacillus* and *Prevotellaceae_UCG-001*. *Lactobacillus* upregulates serum level of PEA while *Prevotellaceae_UCG-001* down-regulates it.

### Alanine, Aspartate and Glutamate Metabolism

L-asparagine (ASN) is synthesized by asparagine synthetase (ASNsynt, EC 6.3.5.4) from aspartic acid (also named aspartate) and L-glutamine (Gln) in nature, and it is the substrate of ASNase (EC 3.5.1.1), which catalyzes its deamidation giving L-aspartic acid and ammonia as reaction products (Covini et al., 2012). Various microorganisms, including commensal intestinal bacteria have the capacity to secrete ASNase; ASNase has been used as an anti-tumor medicine for acute lymphoblastic leukemia and lymphosarcoma, which blocks exogenous ASN supply of tumor cells (Paul et al., 2019). In addition to reports related to antitumor, altered level of L-asparagine/L-aspartic acid has been linked to other diseases (Chen et al., 2020). Alanine, aspartate and glutamate metabolism disorder has also been reported with distinctly changed serum levels of L-aspartic acid and synovial tissue level of L-asparagine in CIA rats (Pang et al., 2021).

We observed a lower serum level of L-aspartic acid and a higher level of L-asparagine in RA rats compared with the control group; the concentration of the two compounds recovered after TFP treatment. Moreover, the abundance of *Lactobacillus* shows positive relation to serum L-asparagine concentration and negative relation to L-aspartic acid concentration, while *Bacteroides* and *Prevotellaceae_UCG-001* showed a negative correlation with serum L-asparagine level. Similar to modulation effects on histamine metabolism, it indicated that Lactobacillus has an adverse correlation with TFP’s regulation of Alanine, aspartate and glutamate metabolism, while *Bacteroides* and *Prevotellaceae_UCG-001* show a beneficial correlation. Notably, Gln, a precursor for the synthesis of asparagine, can be produce from L-glutamate in histamine metabolism. Hence, we infer *Bacteroides* and *Prevotellaceae_UCG-001* mainly affects the generation of L-asparagine *via* L-glutamate/L-glutamine through histamine metabolism.

### Amino Sugar and Nucleotide Sugar Metabolism

While the dysfunction of Amino sugar and nucleotide sugar metabolism has been reported in numerous metabolic abnormalities such as oxidative damage (Salau et al., 2020), dyslipidemia (Huang et al., 2020), and type 2 diabetes (Zhang et al., 2020), we have not found any significant association of this pathway relating to RA in previous researches. In our study, several key metabolites in Amino sugar and nucleotide sugar metabolism are modulated by TFP, which is mediated by *Lactobacillus*, Christensenellace_R-7_group, *Bacteroides*, and *Prevotellaceae_UCG-001*.

As shown in Figure 5, Histidine, Alanine, aspartate and glutamate metabolism, as well as Amino- and nucleotide sugar metabolism, influence each other through L-Glutamate, L-Glutamine, D-Glucosamine-6P, and Fructose 6-phosphate. Considering the aforementioned, we speculate that influences on serum L-histidine/histamine concentrations might be a major contributor to TFP’s therapeutic effects in RA rats, mediated by gut microbiota.

## Conclusion

In summary, our study revealed various ameliorative effects of TFP on RA, including promoting weight gain, improving pathological phenomena in joints, relieving inflammation, and favorably regulating cytokines. These effects most likely relates to the improvement of the dysbiosis that exists in the gut flora, as well as the modulation of Histidine metabolism, Phenylalanine metabolism, Alanine, aspartate and glutamate metabolism, in addition to Amino sugar and nucleotide sugar metabolism (Figure 5). As this is a preliminary in the field, there are a few limitations to it: 1) the dose planning is a little rough without giving an optimal range; 2) we only included pharmacodynamic indexes that were inflammation related, which is not comprehensive; and 3) sequencing and analytical techniques, as well as examination of gut microbiota did not get to the species level, which may result in non-precise conclusions and requires further exploration.

## Data Availability

The datasets presented in this study can be found in online repositories. The names of the repository/repositories and accession number(s) can be found below: PRJNA786950.
