# Supplementary figures and images for: Traditional Tibetan Medicine Twenty-Five Wei’er Tea Pills Ameliorate Rheumatoid Arthritis Based on Chemical Crosstalk Between Gut Microbiota and the Host
Source: Front Pharmacol. 2022 Feb 10;13:828920. doi: 10.3389/fphar.2022.828920 (PMC8867225; doi:10.3389/fphar.2022.828920)

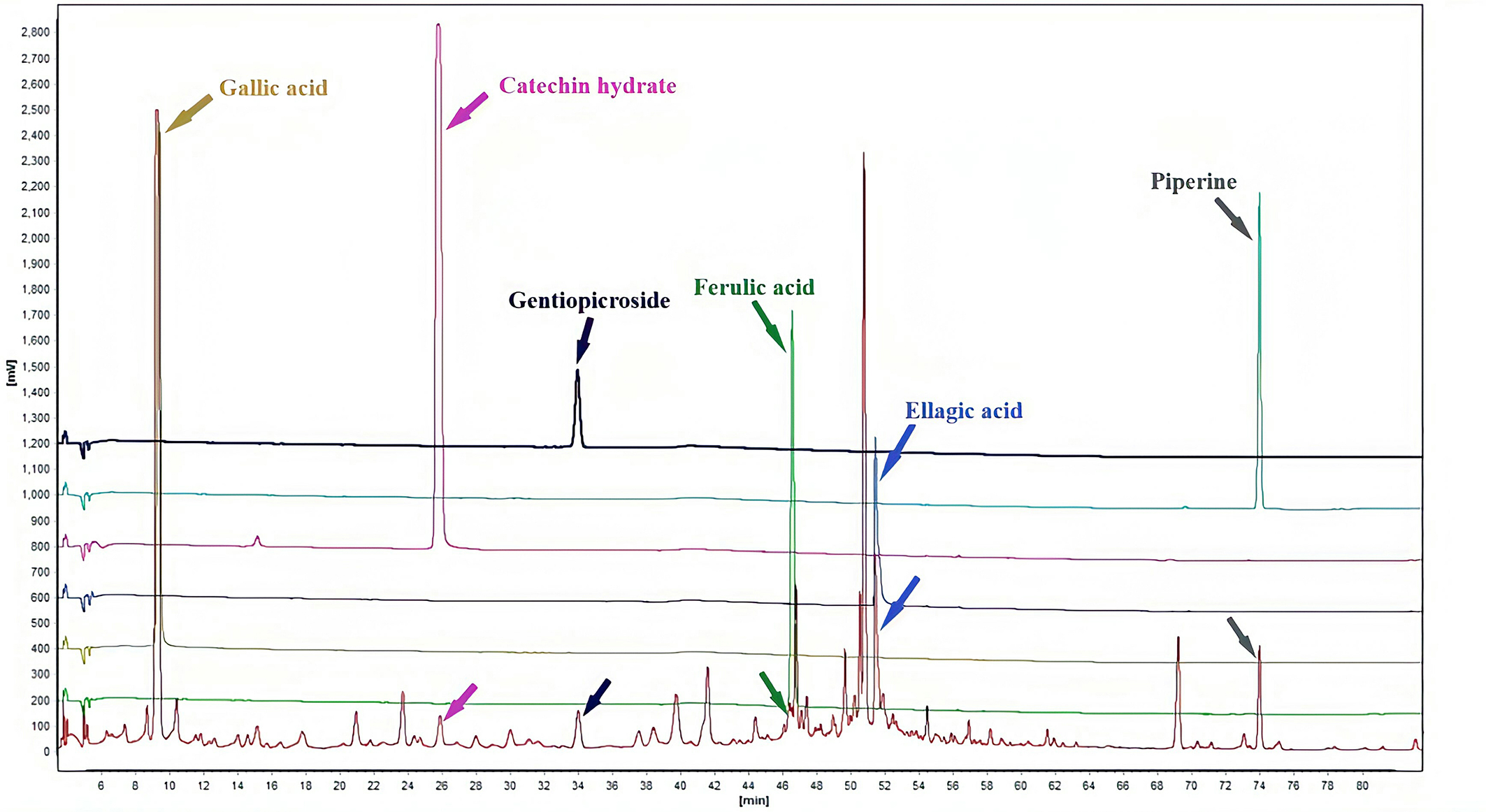

Supplement: Supplementary file 1 [file Image1.JPEG]

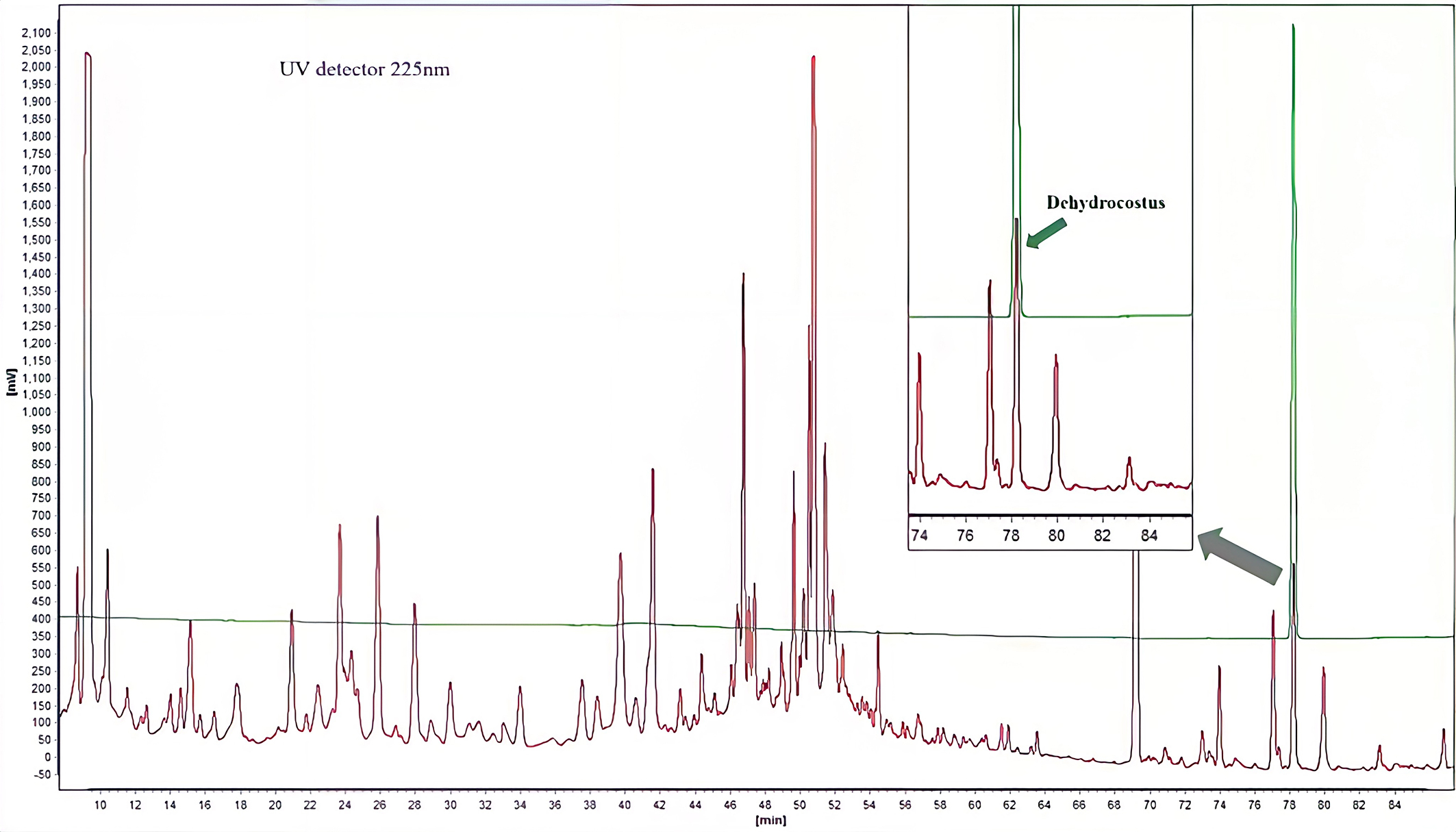

Supplement: Supplementary file 2 [file Image2.JPEG]

Compounds identified in TFP:


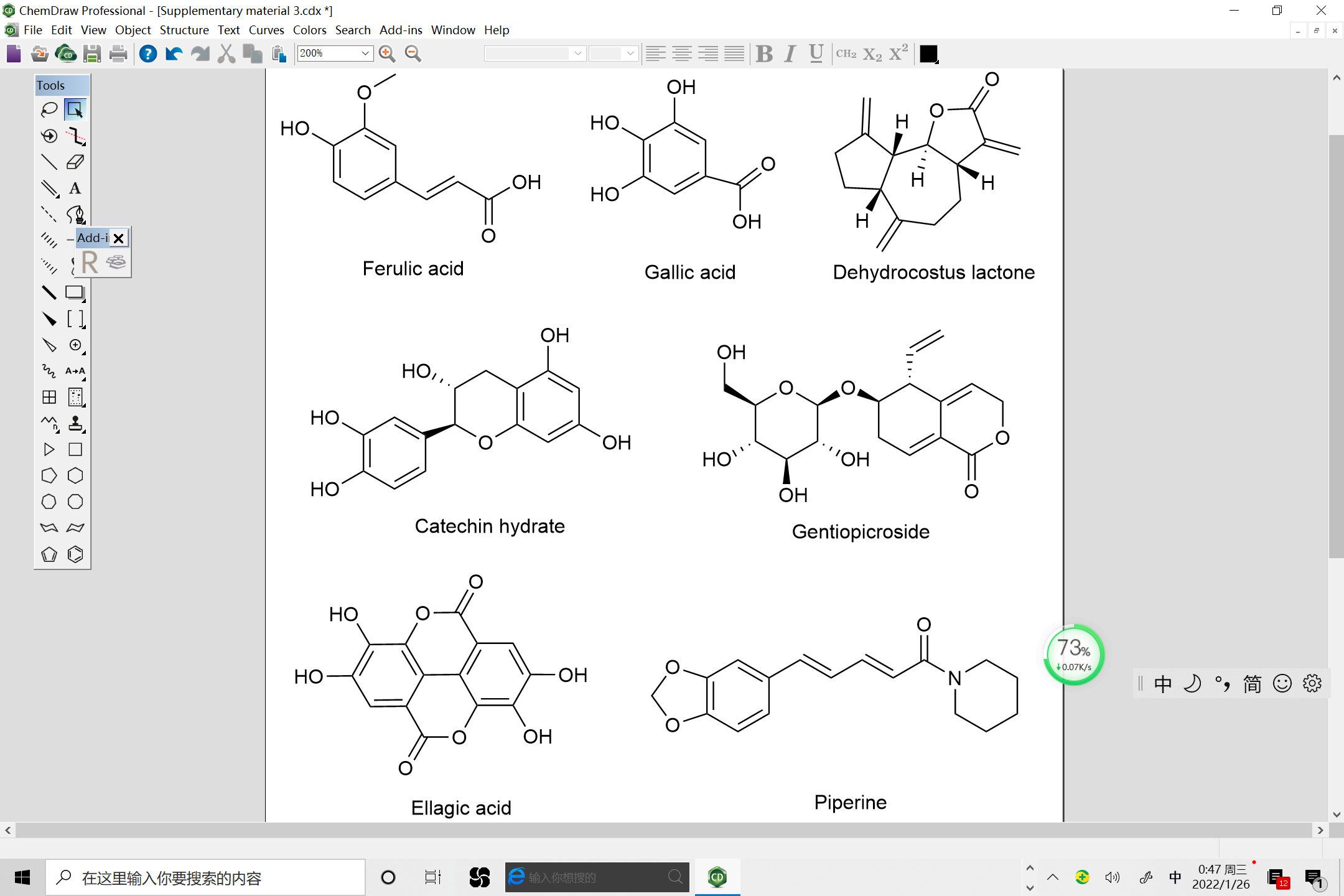

Supplement: Supplementary file 3 [file DataSheet1.docx]
